# Inhibition of primary colon carcinoma growth and liver metastasis by the A3 adenosine receptor agonist CF101

**DOI:** 10.1038/sj.bjc.6601315

**Published:** 2003-10-14

**Authors:** G Ohana, S Bar-Yehuda, A Arich, L Madi, Z Dreznick, L Rath-Wolfson, D Silberman, G Slosman, P Fishman

**Affiliations:** 1 Department of Surgery A/B, Rabin Medical Center, Campus Golda, Sackler Faculty of Medicine Tel-Aviv University, Petach-Tikva 49100, Israel; 2 Laboratory of Clinical and Tumor Immunology, The Felsenstein Medical Research Center, Sackler Faculty of Medicine, Tel-Aviv University, Petach-Tikva 49100, Israel; 3 Department of Pathology, Rabin Medical Center, Campus Golda, Sackler Faculty of Medicine Tel-Aviv University, Petach-Tikva 49100, Israel; 4 Can-Fite Biopharma Ltd, Kiryat-Matalon, Petach-Tikva 49170, Israel

**Keywords:** A3 adenosine receptor, colon carcinoma, natural killer cells, myeloprotection, IL-12, CF101

## Abstract

Adenosine is a purine nucleoside that acts as a regulatory molecule by binding to specific G-protein-coupled A1, A_2A_, A_2B_, and A3 cell surface receptors. We have recently demonstrated that adenosine inhibits tumour cell growth and concomitantly stimulates bone marrow cell proliferation via activation of the A3 adenosine receptor (A3AR). In the present study, we show that a synthetic agonist to the A3AR, CF101, at the low nanomolar concentration range, inhibits HCT-116 human colon carcinoma cell growth. This effect was reversed by the selective A3AR antagonist MRS1523, demonstrating the specificity of the response. CF101 (given orally) was efficacious in inhibiting the development of primary tumours in xenograft and syngeneic models in which mice were inoculated subcutaneously with human HCT-116 or murine CT-26 colon carcinoma cells, respectively. Moreover, CF101 suppressed (50%, *P*<0.01) colon cancer liver metastases in syngeneic mice inoculated to the spleen with CT-26 cells. The mechanism of action entailed upregulation of interleukin-12 production in the CF101-treated groups and potentiation of NK cell activity. In the HCT-116 xenograft model in which a combined therapy of CF101 and 5-fluorouracyl (5-FU) was examined, an additive antitumour effect was demonstrated. Moreover, CF101 prevented the 5-FU-induced myelotoxicity, resulting in normal values of white blood cell and neutrophil counts. We conclude that the A3AR agonist CF101, a small orally bioavailable molecule, exerts systemic anticancer, antimetastatic, and myeloprotective effects in colon carcinoma-bearing mice, and may serve as an adjuvant treatment to enhance the chemotherapeutic index and prevent myelotoxicity.

The realisation that the A3 adenosine receptor (A3AR) may be a new target for cancer therapy is a result of a research that has its roots in a well-recognised clinical phenomenon, that is, the rarity of tumour metastases in striated muscle tissue. It was found that muscle cells secrete small molecules, which inhibit growth of a broad range of different tumour cell lines *in vitro*, such as melanoma, carcinoma, leukemia, and lymphoma (Djaldetti *et al*, 1996; Bar-Yehuda *et al*, 1999). Remarkably, these small molecules induced proliferation of normal cells, such as bone marrow, fibroblasts, and muscle cells. When administered orally to melanoma-bearing mice, these small molecules inhibited the development of lung metastases. Moreover, when given in combination with cyclophosphamide, the molecules enhanced the chemotherapeutic index and induced a myeloprotective effect. Thus, the muscle-derived small molecules show a dual activity entailing inhibition of tumour and stimulation of normal cell growth.

Adenosine was shown to be among those small, muscle-secreted molecules. It is a ubiquitous nucleoside, which is released into the extra cellular environment of metabolically active cells, and was shown, *in vitro*, to possess the aforementioned dual activity (Fishman *et al*, 1998). Adenosine binds to cells through specific A1, A_2A_, A_2B_, and A3 G-protein-associated cell surface receptors, thus acting as a signal transduction molecule by regulating the levels of cAMP and the downstream effector protein kinase A (Stiles, 1990; Linden, 1991). However, oral administration of adenosine to mice did not generate an effect similar to that observed with the muscle-derived small molecules, hence endorsing further research to explore the active component. Pharmacological studies, using antagonists to the different adenosine receptors, revealed that A3AR plays a key role in the adenosine-induced inhibition of tumour cell proliferation, simultaneously stimulating bone marrow cell growth (Fishman *et al*, 2000a, 2000b; Bar-Yehuda *et al*, 2001). Indeed, synthetic agonists to A3AR, such as CF101 and Cl-IB-MECA (Jacobson *et al*, 1995), were found to act similar to adenosine, while having the advantage of being stable, nondegradable, and bioavailable molecules (Fishman *et al*, 2000a, 2000b).

Notably, the differential effect exerted by the A3AR agonists occurred specifically at the nanomolar concentration range, in which exclusive activation of A3AR took place (Fishman *et al*, 2000b, 2001). The mechanism involved in the inhibition of tumour cell growth included a cytostatic effect leading to cell cycle arrest in the G0/G1 phase of the cell cycle. It was further shown that deregulation of the Wnt signalling pathway is involved in the anticancer effect exerted by A3AR agonists (Fishman *et al*, 2002, 2003), hence identifying a potential role for these agonists in the treatment of colon malignancy.

Chemotherapy after primary surgery is recommended for patients with colon carcinoma and nodal involvement to reduce the rate of cancer recurrence and prolong survival. Current therapy is far from being uniformly useful to patients and not without side effects, thus limiting the appropriate dosage and duration of chemotherapy. The stimulatory effect of CF101 on bone marrow cell proliferation was mediated via the induction of G-CSF production (Fishman *et al*, 2000a, 2001; Bar-Yehuda *et al*, 2002). Thus, it became apparent that the A3AR might serve as a target for anticancer and myeloprotective treatments.

Natural cytotoxicity, mediated by NK cells, is believed to play an important role in host anticancer mechanisms. In the colon, neoplastic lesions were found to exist where NK activity was inhibited, while under the influence of normal NK cells, these lesions remained dormant (nonproliferative) (Altmann, 1995). Interleukin-12 (IL-12), previously designated NK cell stimulatory factor (NKSF), is released from antigen-stimulated monocytes, macrophages, and lymphocytes, and mediates several biological activities of T and NK cells, including the induction of INF-*γ* production and enhancement of cell-mediated cytotoxicity (Trinchieri, 1993; Lee *et al*, 1998). IL-12 has been shown to possess anticancer activity in a wide variety of murine tumour models, including carcinoma of the colon (Smyth *et al*, 2000).

In this study we further show that CF101, an A3AR agonist, inhibits the growth of colon carcinoma cells both *in vitro* and *in vivo*, and that in addition to the direct antiproliferative effect an immunomodulatory mechanism is involved.

## MATERIALS AND METHODS

### Drugs

**Figure 1:**
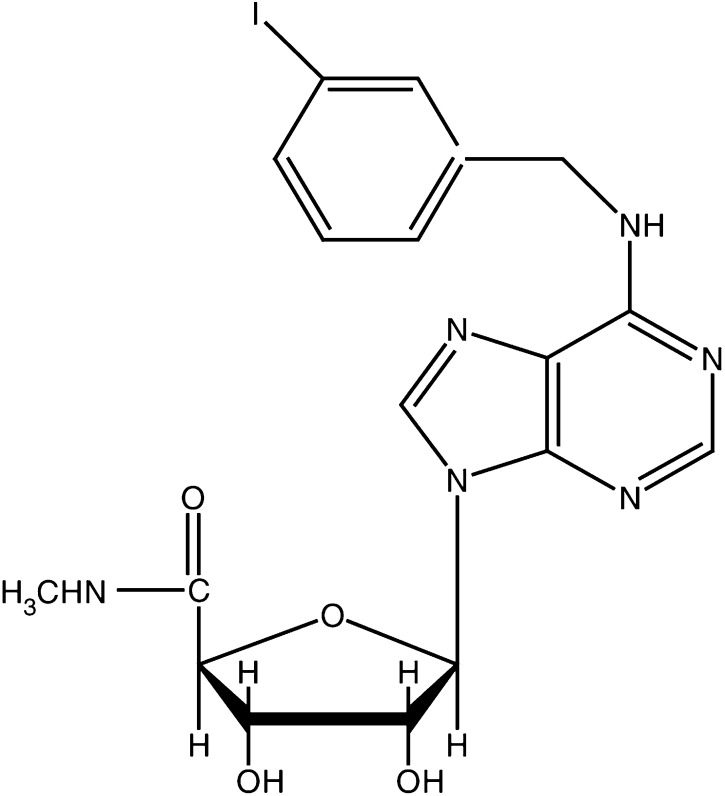
Chemical structure of the synthetic A3AR agonist 1-deoxy-1-[6-[[(iodophenyl)methyl]amino]9H-purine-9-yl]-*N*-methyl-(-D-ribofuranuronamide), CF101.

The A3AR agonist known generically as 1-deoxy-1-amino]-9H-purine-9-yl]-*N*-methyl-(-D-ribofuranuronamide) (CF101), a GMP grade, was synthesised for Can-Fite BioPharma by Albany Molecular Research Inc., Albany, NY, USA (molecular structure is presented in Figure 1

). The A3AR antagonist MRS1523 was purchased from RBI/Sigma. For both reagents, a stock solution of 10 mM was prepared in DMSO and further dilutions in RPMI medium were performed. RPMI, fetal bovine serum (FBS), and antibiotics for cell cultures were purchased from Beit Haemek, Haifa, Israel. The primary antibodies included rabbit polyclonal against murine and human A3AR and the secondary antibodies included anti-rabbit and anti-Goat, respectively, and were purchased from Santa Cruz Biotechnology Inc., CA, USA and served as primary antibodies.

The chemotherapy agent 5 fluorouracil (5-FU) was purchased from ABIC (Israel).

### Tumour cells

HCT-116 human and CT-26 murine colon carcinoma cells were purchased from the American Type Tissue Culture Collection (ATCC, Rockville, MD, USA). Cells were maintained in RPMI medium supplemented with 10% FBS, 200 mM glutamine, 100 U ml^−1^ penicillin, and 100 *μ*g ml^−1^ streptomycin. Cells were transferred to a freshly prepared medium twice weekly. For studies in which we used serum-starved cells, FBS was omitted from the cultures for 18 h and the experiment was carried out on monolayers of cells in RPMI medium supplemented with 1% FBS in a 37°C, 5% CO_2_ incubator.

### Examination of A3AR protein expression in colon carcinoma cells by Western Blot (WB) analysis

To detect the protein expression level of A3AR, HCT-116 human colon carcinoma or CT-26 murine cells (5 × 10^4^ ml^−1^) were incubated in the presence and absence of CF101 (10 nM) for 15 min at 37°C with 1% FBS. Cell samples were rinsed with ice-cold PBS and transferred to ice-cold lysis buffer (TNN buffer, 50 mM Tris buffer pH 7.5, 150 mM NaCl, NP-40). The trypsinised cells were washed again with ice-cold PBS, harvested by centrifugation, and subjected to lysis in TNN buffer. Cell debris was removed by centrifugation for 10 min, at 7500 **g**. The supernatant was utilised for WB analysis. Protein concentrations were determined using the Bio-Rad protein assay dye reagent. Equal amounts of the sample (50 *μ*g) were separated by SDS–PAGE, using 12% polyacrylamide gels. The resolved proteins were then electroblotted onto nitrocellulose membranes (Schleicher & Schuell, Keene, NH, USA). Membranes were blocked with 1% bovine serum albumin and incubated with the relevant primary antibody (dilution 1 : 1000) for 24 h at 4°C. Blots were then washed and incubated with the secondary antibody for 1 h at room temperature. Bands were recorded using BCIP/NBT color development kit (Promega, Madison,W1, USA). Data presented in the figure are representative of at least four different experiments.

### Cell proliferation assay

[^3^H]thymidine incorporation assay was used to evaluate cell growth. HCT-116 colon carcinoma cells (5 × 10^4^ ml^−1^) were incubated with CF101 at a concentration of 0.1, 1, and 10 *μ*M, in 96-well microtitre plates for 24 h. To test whether CF101 exerted its effect on tumour cells through specific binding to the A3AR, MRS1523 (0.1 *μ*M), an antagonist to the A3AR, was added to the cell cultures 30 min prior to CF101 introduction. Cultures of HCT-116 cells that were incubated in the presence of MRS-1523 only, served as controls. For the last 18 h of incubation, each well was pulsed with 1 *μ*Ci [^3^H]thymidine. Cells were harvested and the [^3^H]thymidine uptake was determined in an LKB liquid scintillation counter (LKB, Piscataway, NJ, USA). These experiments were repeated at least four times.

### *In vivo* studies

All the experiments were performed in accordance with the guidelines established by UKCCCR guidelines (Workman *et al*, 1998) and the Institutional Animal Care and Use Committee at the Rabin Medical Center, Petach Tiqva, Israel.

In this set of experiments, we tested the effect of CF101 on the growth of human HCT-116 and murine CT-26 colon carcinoma cells. For the HCT-116 model, cells (1.2 × 10^6^) were subcutaneously injected to the flank of Nude/BalbC mice. Mice were divided into four groups and treatment was initiated 1 day after tumour inoculation. Each group contained 10 mice and the experiment was repeated three times.

CF101 dose was chosen based on the PK analysis after oral administration of CF101 to mice and the assumption that absorption of the drug is linearly related to the administered dose. A dose of 10 *μ*g kg^−1^ yielded a maximal drug concentration of about 4 nM, in which all receptors are expected to be bound with the drug (*K*_*i*_=0.4 nM) (unpublished data).

The following protocol was utilised:

1. control group – vehicle only.
2. 10 *μ*g kg^−1^ body weight CF101, given orally once daily (initiated 7 days after tumour inoculation),
3. chemotherapy – one cycle of intraperitoneal 5-FU at 20 mg kg^−1^, given once a day for five consecutive days (initiated 1 day after tumour inoculation),
4. 5-FU+10 *μ*g kg^−1^ body weight CF101 (as detailed above).

Tumour size (width (*W*) and length (*L*)) was measured every 4 days and was calculated according to the following formula:


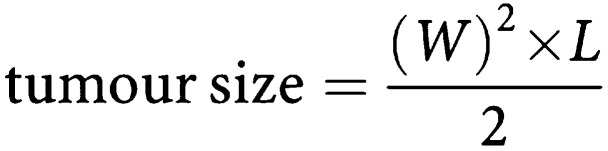


We further compared the response of each treatment *vs* control by calculating the percentage of tumour growth inhibition, at each time point, according to the following formula:


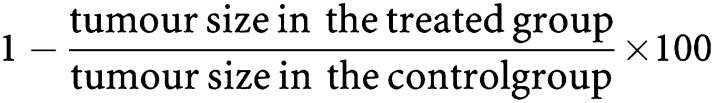


The study was terminated when tumour size in the CF101-treated group reached the EC_50_.

To test the myeloprotective effect of CF101, blood samples were withdrawn 48 h after the initiation of CF101 treatment.

For the CT-26 primary colon carcinoma model, cells (1x10^6^) were subcutaneously injected to the flank of syngeneic Balb/C mice. Treatment was initiated 1 day after tumour inoculation and mice were killed after 15 days.

For the CT-26 liver metastasis model, syngeneic Balb/C mice were anaesthetised with ketamine (22  mg kg^−1^) and xylazine (10  mg kg^−1^). After prepping with betadine, left upper quadrant laparotomy and splenic exteriorisation were performed. Using a 27-gauge needle, 50 *μ*l of the tumour cell suspension (0.5 × 10^6^ cells ml^−1^ PBS) was injected beneath the splenic capsule. The spleen was returned to the peritoneal cavity followed by two-layer closure of the peritoneum and skin using 6-0 nylon sutures. The mice were treated daily orally with 10 *μ*g kg^−1^ CF101 or the vehicle (each group contained 10 mice and the experiment was repeated three times). Treatment was initiated 1 day after tumour inoculation and mice were killed after 12 days. Liver metastasis was evaluated by a score ranging from 0 to 4, based on metastasis load.

### Blood cell counts

White blood cell (WBC) counts were carried out in a Coulter counter and differential cell counts were performed on smear preparations stained with May–Grunvald–Giemsa solution.

### *Ex vivo* NK activity

To test NK cell activity of splenocytes derived from CF101- and vehicle-treated HCT-116 tumour-bearing mice, the standard 4 h release assay was used. YAC lymphoma cells served as targets. Splenocytes (1 × 10^6^) were cultured in 96-well plates and resuspended in RPMI containing 10% FBS. The YAC lymphoma cells were labelled with 100 *μ*Ci of Na_2_O_4_ at 37°C for 1 h. cells (2 × 10^4^) were then resuspended and mixed with the effector cells at an E : T ratio of 50 : 1, in a volume of 200 *μ*l. After 4 h of incubation at 37°C in 5% CO_2_, plates were centrifuged and supernatants were counted in a gamma counter (LKB). NK cytotoxicity was calculated using the following equation:


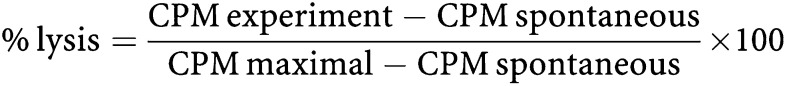


Spontaneous and maximal counts per minute (CPM) were determined by measuring the supernatant's CPM of target cells (alone or in the presence of 1% SDS). The spontaneous release was below 10% of the maximal release throughout the experiments.

### IL-12 analysis in serum sapmles

Serum samples for IL-12 analysis were obtained from HCT-116 tumour-bearing mice previously treated or untreated with the drug and assayed by a commercial murine ELISA kit of R&D systems, Minneapolis, MN, USA.

### Statistical analysis

The efficacy of the various agents *in vitro* and *in vivo* was evaluated using the Student's *t*-test. The criterion for statistical significance was *P*<0.05.

## RESULTS

### A3AR expression in HCT-116 and CT-26 colon carcinoma cells

**Figure 2:**
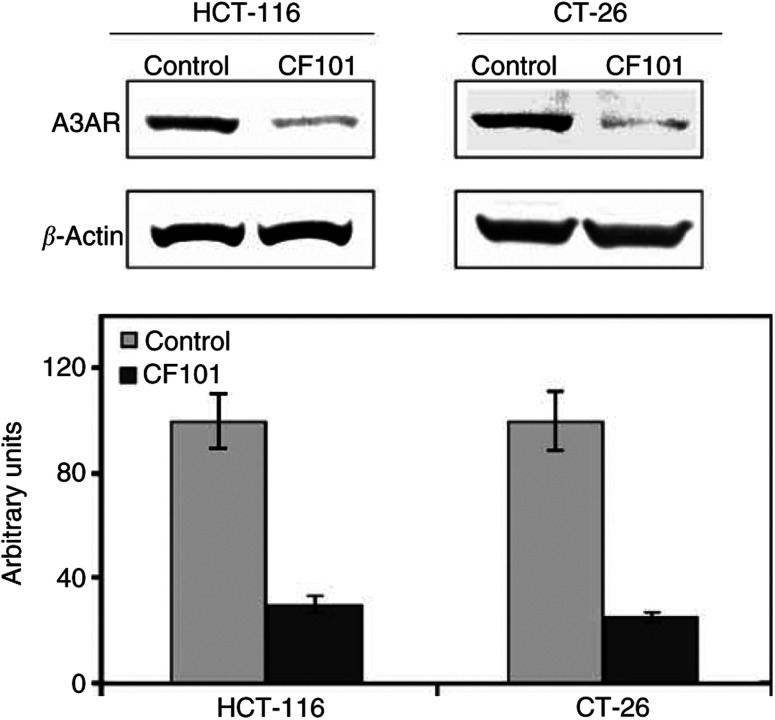
Protein expression level of A3AR in HCT-116 and CT-26 colon carcinoma cells. Cells (5 × 10^4^ ml^−1^) were incubated for 15 min at 37°C with and without 10 nM CF101. High A3AR expression level is seen in the control *vs* downregulation of receptor expression, indicating that response to the agonist takes place after 15 min.

A3AR was highly expressed in HCT-116 and CT-26 cells. Upon treatment with CF101, the receptor level was downregulated indicating the cell response to the agonist. The level of expression was quantified based on a comparison to the housekeeping gene *β*-actin (Figure 2

).

### CF101 inhibits cell proliferation *in vitro*

**Figure 3:**
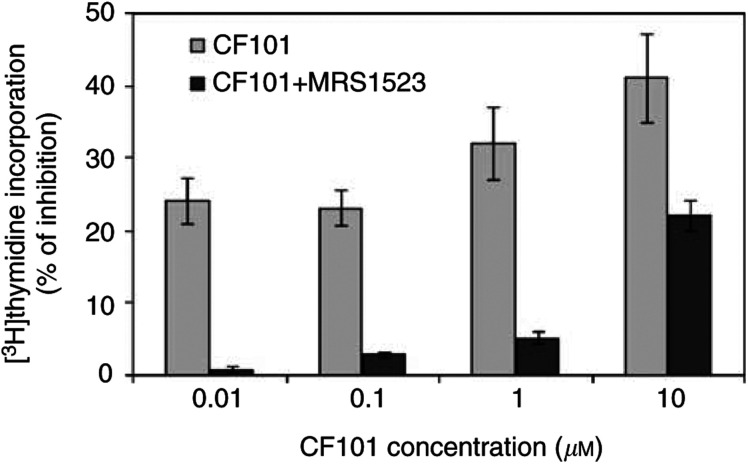
CF101 inhibits the proliferation of HCT-116 human colon carcinoma cells *in vitro*. Cell proliferation was measured by the [^3^H]thymidine incorporation assay. The introduction of MRS1523 counteracted most of the inhibitory effect that was exerted by CF101.

CF101 exerted a dose-dependent inhibitory effect on the growth of HCT-116 cells *in vitro*, at the nanomolar and micromolar concentration range (*P*<0.002, for all concentrations). The A3AR antagonist MRS1523 counteracted most of the inhibitory effect of CF101 at the nanomolar concentrations (*P*<0.001, for all the concentrations), whereas at the 10 *μ*M concentration, the inhibitory effect was not fully blocked by the antagonist (CF101 *vs* MRS1523, *P*<0.05) (Figure 3

). In an additional set of experiments, we tested the effect of higher CF101 dosages on the proliferation of HCT-116 cells. EC50 was detected at a drug concentration of 30 *μ*M; however, this effect was not counteracted by MRS1523. These results demonstrate that at low CF101 concentrations, the response is exclusively A3AR mediated. However, at the micromolar range, additional adenosine receptors may be activated, which are responsible for the tumour growth proliferation inhibition.

### CF101 inhibits the development of primary tumour and liver metastasis *in vivo*

**Figure 4:**
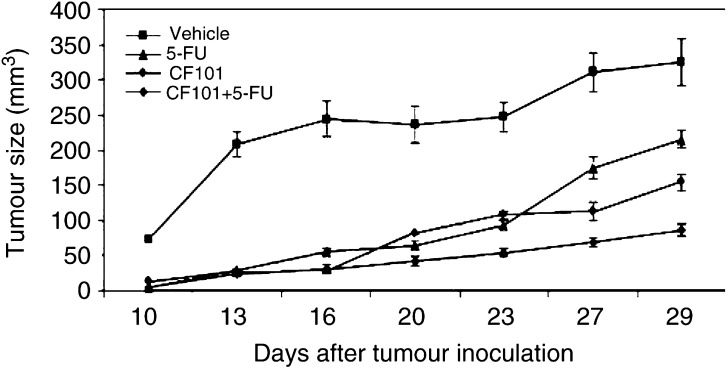
Effect of CF101 alone or in combination with 5-FU on the growth of HCT-116 human colon carcinoma cells in nude mice. Tumour cells (1.2 × 10^6^) were subcutaneously injected to the flank of nude mice. The following treatment modalities were applied: 5-FU (20  mg kg^−1^, given once a day for 5 days, initiated 1 day after tumour inoculation); 10 *μ*g kg^−1^ CF101 daily orally, initiated 7 days after tumour inoculation; combination of 5-FU and CF101 as detailed above for each treatment alone and vehicle. Tumour size was measured every 4 days.

In these experiments, we evaluated the inhibitory effect of CF101, given as a monotherapy or in combination with 5-FU, to inhibit the growth of flank human HCT-116 colon carcinoma tumours. Tumour size was markedly inhibited following treatment with CF101 or 5-FU (*P*<0.0001 and <0.0001, respectively), and the combined therapy yielded an additive effect (*P*<0.0001) (Figure 4

). Table 1

**Table 1:** % of tumour size inhibition at various time points

**Day**	**5-FU**	**CF101**	**CF101+5-FU**
10	95.27	81.21	92.95
13	86.06	86.9	87.86
15	77.57	88.42	87.86
20	72.85	65.53	82.09
23	62.27	56.49	78.26
27	43.75	63.79	78.02
29	33.77	52.55	73.43

Effect of CF101 alone or in combination with 5-FU on the growth of HCT-116 human colon carcinoma cells in nude mice. Tumour cells (1.2 × 10^6^) were subcutaneously injected to the flank of nude mice. The following treatment modalities were applied: 5-FU (20 mg kg^−1^, given once a day for 5 days, initiated 1 day after tumour inoculation), 10 *μ*g kg^−1^ CF101 daily orally, initiated 7 days after tumour inoculation, combination of 5-FU and CF101, as detailed above for each treatment alone and vehicle. Tumour size was measured every 4 days.

**Figure 5:**
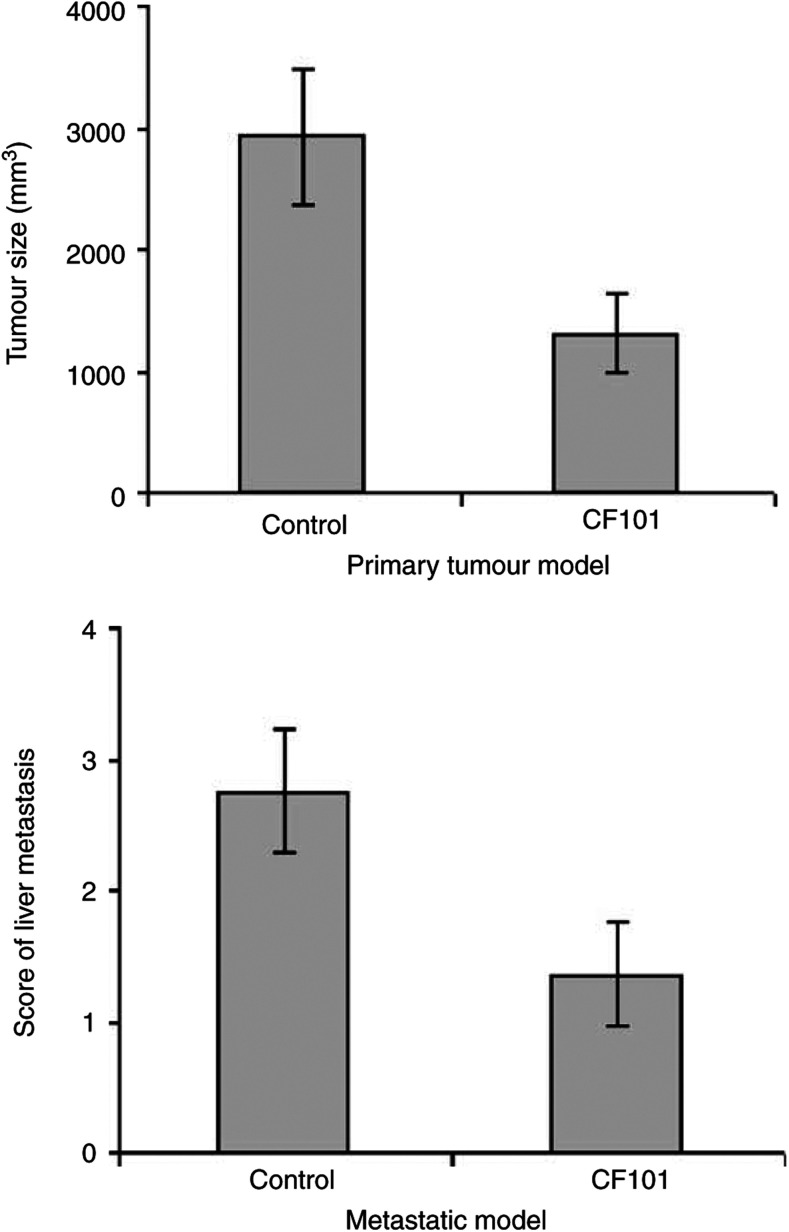
Effect of CF101 on the development of primary tumour and liver metastasis in CT-26 colon carcinoma-bearing mice. For the primary tumour model CT-26 cells were subcutaneously injected to the flank of nude mice, and for the liver metastatic model CT-26 cells were injected beneath the spleen capsule. For both models, treatment was initiated 1 day after tumour cell inoculation and included daily oral administration of CF101 (10 *μ*g kg^−1^) till the mice were killed.

. summarises the percentage of tumour size inhibition at various time points, demonstrating that tumour growth inhibition in the combined treatment yielded the maximal effect. We further evaluated the inhibitory effect of CF101 on the development of primary and liver metastasis in mice inoculated with CT-26 murine colon carcinoma cells. A marked inhibition in the development of the flank tumours (*P*<0.01) and liver metastasis (*P*<0.01) was observed in the CF101-treated mice (Figure 5

).

### CF101 acts as a myeloprotective agent

**Figure 6:**
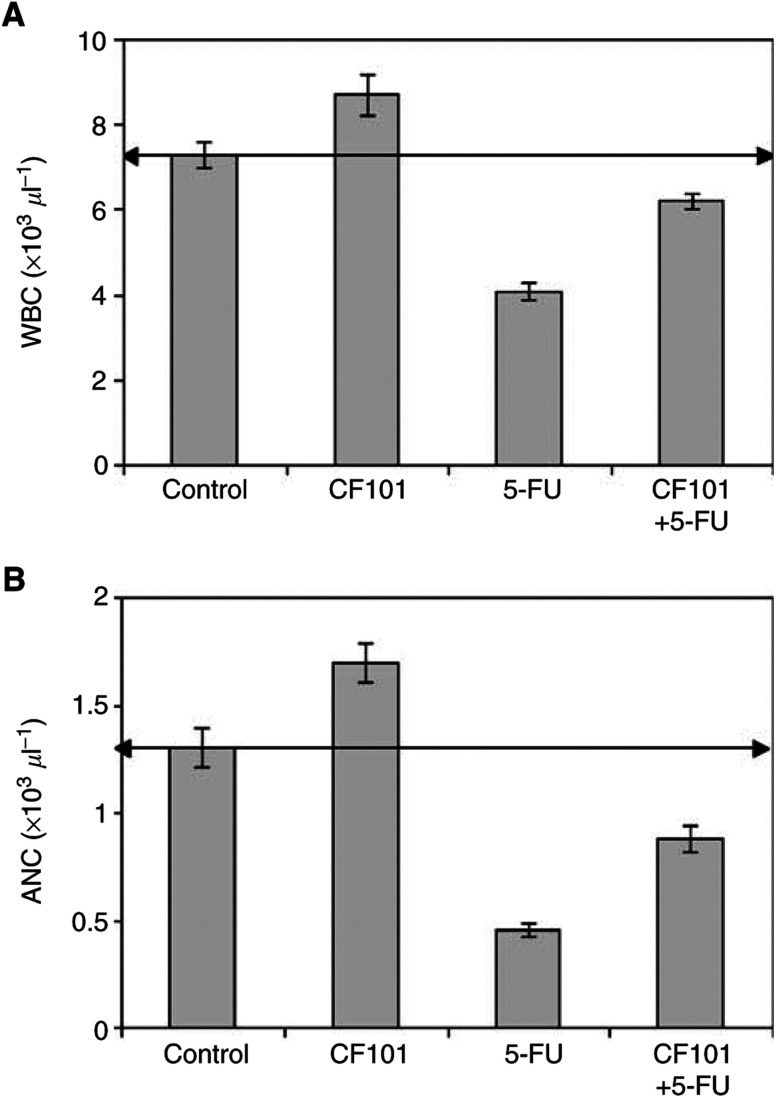
*In vivo* effect of CF101 on the number of WBC and ANC in mice treated with 5-FU (20  mg kg^−1^, given once a day for 5 days). Chemotherapy alone decreased the number of WBC and ANC of neutrophils. CF101, administered orally after chemotherapy, increased the number of WBC (**A**) and percentage of neutrophils (**B**) to almost normal values.

Mice treated with 5-FU exhibited a decline in the number of peripheral blood leucocytes and absolute neutrophil counts (ANC). Administration of CF101 following chemotherapy increased the number of WBC (Figure 6A

) and restored the percentage of neutrophils (*P*<0.001 and <0.01, respectively) (Figure 6B).

### CF101 acts as an immunomodulatory agent

**Figure 7:**
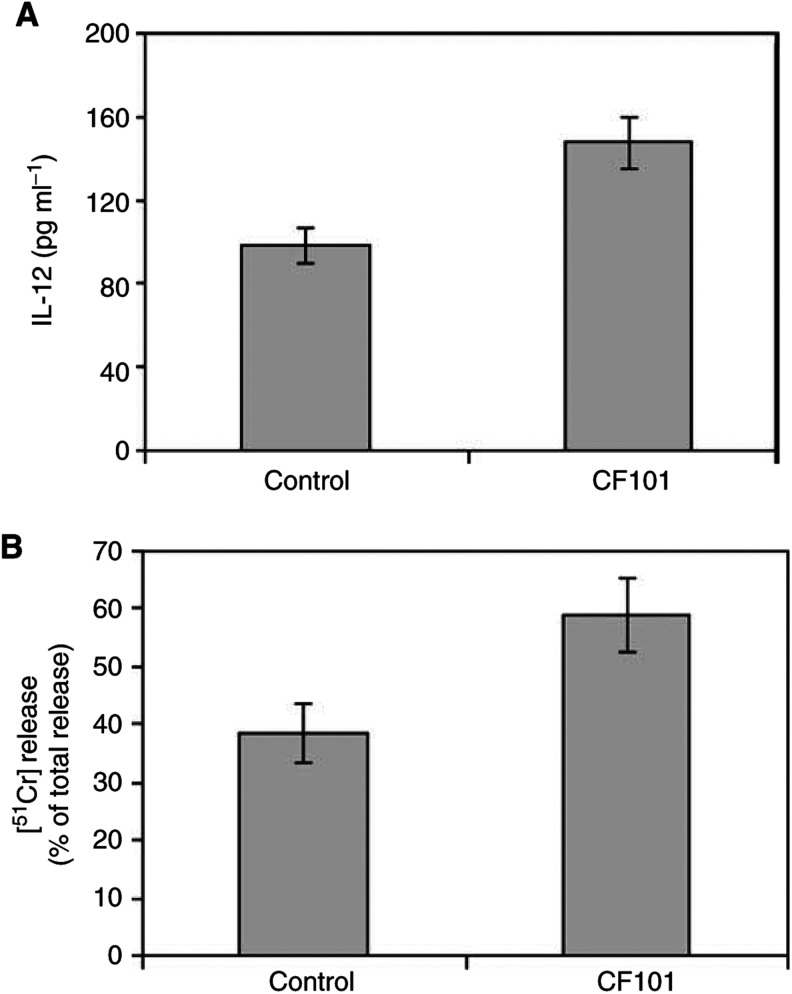
Effect of CF101 on serum IL-12 level and NK activity in HCT-116 bearing mice. Tumour cells (1.2 × 10^6^) were subcutaneously injected to the flank of nude mice. The mice were treated daily orally with 10 *μ*g kg^−1^ CF101. Mice were killed on day 29, serum was evaluated for IL-12 level utilising ELISA and NK activity of splenocytes was determined by the [^51^Cr]-release assay.

To test whether the inhibitory activity of CF101 is also mediated by an indirect immunomodulatory effect, we tested IL-12 serum level and NK cell activity in the HCT-116 tumour-bearing mice. CF101 treatment elevated IL-12 serum level and potentiated NK cell activity (*P*<0.05 for both) (Figure 7A,B

).

## DISCUSSION

This study shows that orally administered CF101, an A3AR agonist, possesses anticancer activity against HCT-116 human and CT-26 murine colon carcinoma, while having the ability to protect against chemotherapy-induced myelotoxicity.

CF101 exerted *in vitro* a dose-dependent inhibitory effect on the growth of HCT-116 human colon carcinoma cells at the low *μ*M concentration range. The MRS1523 antagonist reversed this inhibitory effect at the low micromolar concentrations, demonstrating that the inhibitory effect of CF101 was A3AR mediated. At the higher concentration (10 *μ*M) the activity of CF101 was only partially counteracted by MRS1523, while at 100 *μ*M there was no effect of the antagonist on the level of the growth inhibition induced by the CF101, demonstrating that additional adenosine receptor may be involved in the response.

We thus concluded to utilise *in vivo* only low concentrations of CF101, in order to test exclusively the effect on A3AR activation. Oral administration of CF101 to nude mice resulted in the inhibition of HCT-116 human colon carcinoma growth. Moreover, an additive inhibitory effect was observed when CF101 was administered in combination with 5-FU. The efficacy of CF101 *in vivo* was demonstrated in additional primary and metastatic colon carcinoma models. CF101 suppressed the development of the primary tumour and the liver metastases of colon carcinoma CT-26 cells. It is suggested that CF101 effect is tumour nonspecific and that a general systemic mechanism is induced. Indeed, in this study, we show that CF101 increased IL-12 production and potentiated the activity of NK cells. Splenocytes obtained from mice treated orally with CF101 exhibited an increased NK cell activity when incubated *ex vivo* with target cells. It is well established that NK cells play a role in the regulation of tumour growth (Miller, 2001) and that the activation of endogenous NK cells in a tumour-bearing host may be therapeutically beneficial.

Dibutyryl cAMP or forskolin (both activate adenylyl cyclase and elevate cAMP level in cells) was reported to inhibit the cytolytic activity of NK cells against certain tumour target cells (Hall *et al*, 1983). Activation of the A3AR, which inhibits adenylyl cyclase activity and decreases cAMP levels, may thus lead to the activation of NK cells.

Link *et al* (2000) showed that a specific A2a receptor agonist inhibited the production of IL-12 in whole blood and monocyte cultures. In addition, *in vivo* studies by Hasko *et al* (1998) in BALB/c mice, pretreated intraperitonealy with IB-MECA (0.2–0.5  mg kg^−1^), demonstrated a decrease in the lipopolysaccharide-induced plasma levels of interleukin-12 and interferon-gamma. It is suggested that high CF101 doses may activate the A2A receptor resulting in IL-12 downregulation, whereas low doses, as was used in this study, exclusively activate the A3AR and will results in IL-12 upregulation and potentiation of NK cell activity.

In the present study, CF101, *in vivo*, counteracted the myelotoxicity induced with 5-FU by increasing the number of WBCs and neutrophils. Based on our previous data, we suggest that the chemoprotective effect of CF101 was mediated by the induction of G-CSF production (Fishman *et al*, 2001b). G-CSF is clinically used to reduce the length of neutropenia following chemotherapy and bone marrow transplantation. It stimulates the proliferation and differentiation of hematopoietic progenitors and also controls the functional activities of neutrophils and macrophages (Ikebuchi *et al*, 1988; Itoh *et al*, 1992). Our results are supported by those of Pospisil *et al* (1993), 1995, 1998), who showed that administration of adenosine monophosphate (AMP) combined with dypyridamole and G-CSF, prior to radiation, led to a radioprotective effect by the stimulation of haematopoiesis in the bone marrow and spleen of treated mice. The chemoprotective feature of the A3AR agonists is associated with other cytoprotective characteristics of these compounds. A3AR activation by low agonist concentration has been found to mediate functions, such as cerebroprotective activity following chronic administration of IB-MECA to gerbils with cerebral ischaemia (von Lubitz *et al*, 1994), cardioprotective activity during prolonged stimulated ischaemia by rescuing injured myocytes (Liu *et al*, 1994), and an anti-inflammatory effect (Bowlin *et al*, 1997).

Taken together, activation of the A3AR induces a dual effect in colon carcinoma-bearing mice, that is, the induction of anticancer activity concomitantly with a myeloprotective effect. The anticancer activity was shown to be attributed to direct antiproliferative effect and to an indirect effect manifested by upregulation of IL-12 and NK cell activity. The above-mentioned unique properties of CF101 suggest a potential role for the A3AR agonists in the combat of colon cancer.
